# Malaria diagnosis and treatment practices following introduction of rapid diagnostic tests in Kibaha District, Coast Region, Tanzania

**DOI:** 10.1186/1475-2875-12-293

**Published:** 2013-08-26

**Authors:** Marycelina Mubi, Deodatus Kakoko, Billy Ngasala, Zul Premji, Stefan Peterson, Anders Björkman, Andreas Mårtensson

**Affiliations:** 1 Department of Medicine, Malaria Research, Karolinska Institutet, Stockholm, Sweden; 2 Department of Parasitology and Medical entomology, School of Public Health and Social Sciences, Muhimbili University of Health and Allied Sciences, Dar es Saalam, Tanzania; 3 Department of Behavioural Sciences, School of Public Health and Social Sciences, Muhimbili University of Health and Allied Sciences, Dar es Saalam, Tanzania; 4 Department of Public Health Sciences, Division of Global Health (IHCAR), Karolinska Institutet, Stockholm, Sweden; 5 IMCH, Department of Women’s and Children’s health, Uppsala University, Uppsala, Sweden; 6 School of Public Health, Makerere University, Kampala, Uganda

**Keywords:** Diagnosis, Malaria, Prescription practices, Health worker perceptions, Tanzania

## Abstract

**Background:**

The success of the universal parasite-based malaria testing policy for fever patients attending primary health care (PHC) facilities in Tanzania will depend highly on health workers’ perceptions and practices. The aim of this study was, therefore, to assess the present use of malaria diagnostics (rapid diagnostic tests (RDTs) and microscopy), prescription behaviour and factors affecting adherence to test results at PHC facilities in Kibaha District, Coast Region, Tanzania.

**Methods:**

Exit interviews were conducted with fever patients at PHC facilities and information on diagnostic test performed and treatment prescribed were recorded. Interviews with prescribers to assess their understanding, perceptions and practices related to RDTs were conducted, and health facility inventory performed to assess availability of staff, diagnostics and anti-malarial drugs.

**Results:**

The survey was undertaken at ten governmental PHC facilities, eight of which had functional diagnostics. Twenty health workers were interviewed and 195 exit interviews were conducted with patients at the PHC facilities. Of the 168 patients seen at facilities with available diagnostics, 105 (63%) were tested for malaria, 31 (30%) of whom tested positive. Anti-malarial drugs were prescribed to all patients with positive test results, 14% of patients with negative results and 28% of patients not tested for malaria. Antibiotics were more likely to be prescribed to patients with negative test results compared to patients with positive results (81 *vs* 39%, p < 0.01) and among non-tested compared to those tested for malaria (84 *vs* 69%, p = 0.01). Stock-outs of RDTs and staff shortage accounted for the low testing rate, and health worker perceptions were the main reason for non-adherence to test results.

**Conclusions:**

Anti-malarial prescription to patients with negative test results and those not tested is still practiced in Tanzania despite the universal malaria testing policy of fever patients. The use of malaria diagnostics was also associated with higher prescription of antibiotics among patients with negative results. Strategies to address health system factors and health worker perceptions associated with these practices are needed.

## Background

Global efforts to control malaria have recently led to reduction in the overall disease burden, with mortality due to malaria estimated to have declined from 985,000 in 2000 to 660,000 deaths in 2010 [1]. Lower malaria prevalence has therefore been reported from several sub-Saharan African countries, including Tanzania [2,3]. In spite of this decrease, malaria remains a leading public health problem in many areas of sub-Saharan Africa, especially in children under five years of age who remain most at risk of frequent and severe malaria episodes with high mortality.

The introduction of artemisinin-based combination therapy (ACT) has improved malaria case management substantially. However, development and spread of ACT resistance may have drastic consequences for the recent malaria control achievements. For this reason it has become increasingly important to change from symptom-based presumptive treatment to parasitolo gical confirmation of malaria infection before initiation of anti-malarial treatment. The use of parasite-based diagnosis will allow better targeting of anti-malarial drugs, and also provide an opportunity for other causes of fever to be identified and appropriately treated [4]. Therefore, WHO now recommends that anti-malarial treatment be confined to laboratory confirmed cases only [5], and the availability of rapid diagnostic tests (RDTs) offers a good opportunity to extend parasitological confirmation of malaria infection to peripheral areas where quality microscopy cannot be guaranteed [6,7].

The advantages offered by RDTs are well known [6,8,9] although adherence to test results has been a challenge. Several studies have reported poor adherence to RDT negative test results [10-12], and underutilization of testing facilities [13,14], while other studies have shown opposite results with high utilization of RDTs and good adherence to negative test results [15-19]. Importantly, in the latter studies, overall health outcome of fever patients also improved after RDT use, probably because of identification and management of non-malarial fevers. The discordant findings on adherence to RDT results underscore the need to identify and address factors for non-adherence in order to optimize the impact of RDTs.

It has been shown that perceptions of health service providers are important in determining their prescription practices [20,21]. In some instances, health service providers have reported “feeling under pressure” to prescribe anti-malarial drugs to patients with RDT negative results, which suggests that patient perceptions and attitudes may also affect prescription behaviour [20,22].

The National Malaria Control Programme (NMCP) in Tanzania has just completed rolling out malaria RDT to ensure universal parasite-based diagnosis of all fever patients attending primary health care (PHC) facilities. With the conflicting findings of the impact of RDT use in mind it is critical to identify factors affecting compliance in the local setting in order to develop context specific interventions and maximize the benefits of RDT roll out. This study therefore aimed to assess the present use of malaria diagnostics at PHC facilities in Kibaha District, Tanzania, and health workers’ as well as patients’ perceptions about malaria diagnosis using RDT. The study also addressed prescription behaviour and identification of factors affec ting adherence to test results.

## Methods

### Setting of the study

This cross-sectional survey was conducted in Kibaha District, Coast Region, Tanzania. In this region malaria transmission is perennial, with peaks towards the end of the long and short rains, that is May-July and December-January, respectively. *Plasmodium falciparum* is the dominant malaria species. Artemether-lumefantrine is first-line treatment for uncomplicated malaria since 2006. RDT implementation, using combo tests with a combination of histidine-rich protein 2 (HRP2) and parasite lactate dehydrogenase (pLDH) to detect both falciparum and non-falciparum species, was introduced in November 2009.

### Sample and sampling process

At the time of the study there were 32 existing public health facilities in Kibaha District (urban and rural). The inclusion criteria were health facilities at peripheral level offering services to the general public. The exclusion criteria were facilities at higher levels of care, and those belonging to government institutions, private or religious organisations. A total of seven health facilities were excluded from sampling due to the higher level of health care (one regional hospital and one health centre/district hospital) or belonging to government and religious institutions (five dispensaries) serving special groups of patients. From the remaining 25 eligible facilities, ten were selected by ballot. One of these facilities was a health centre with in-patient facilities, while the remaining nine were dispensaries offering out-patient services only.

Health service providers were interviewed after giving verbal informed consent. Exit interviews were held on two consecutive days at each study site with all patients aged three months and above, and/or their guardians, presenting with fever or history of fever after giving their verbal consent. The interviews were conducted by two postgraduate students from the University.

### Data collection

Health care providers were interviewed using questionnaires with both open and closed questions to collect information on their knowledge and perception, and prescription behaviour in relation to malaria RDTs. Exit interviews were also conducted with fever patients or, in case of children, their caretakers to collect information about their knowledge and attitudes towards malaria testing. Patients’ presenting symptoms, malaria diagnostic tests performed, results, and prescribed drugs were retrieved from their case notes and recorded onto case record forms (CRF) developed for the purpose of this study. In addition a health facility inventory was undertaken to collect information on staffing, availability of supplies and equipment, including RDTs, microscopes and anti-malarial drugs, as well as routine statistics on total out-patient attendance, patients with fever, patients tested for malaria, patients with positive results and anti-malarial drug prescriptions over a 12-month period (August 2010-July 2011).

### Data analysis

Data were entered using EpiData 3.1 and statistical analysis was done using SPSS 17 statistical software. Descriptive analysis of the availability of malaria diagnostics and ACT was assessed at health facility level, while exposure to training and supervision was analysed at health worker level. To assess the performance of the new RTD policy in terms of testing rates and anti-malarial prescription rates, analysis was at patient level and the X^2^ test was used, with a p-value of <0.05 considered statistically significant. Adherence to malaria test results was analysed using logistic regression. Analysis of both health workers’ and patients’ knowledge and perceptions was done after coding the open-ended responses into categories.

### Ethical approval

Ethical approval for the study was provided by the Research and Publication Committee of the Muhimbili University of Health and Allied Sciences. Verbal informed consent was obtained from patients or their caretakers and health workers involved in the study.

## Results

### Profile of health facilities, health workers and patients

The ten selected study sites (nine dispensaries and one health centre) had in total 114 staff members, of whom 55 (48%) were nursing staff, 30 (26%) had clinical training and 29 (25%) belonged to other cadres (laboratory personnel, health officers and medical attendants). The health centre with in-patient facilities had 15 clinicians, whereas the remaining nine facilities had between one and three clinicians.

Nine of ten health facilities reported RDT stock-outs at some point since their introduction. Eight study sites were equipped with parasite-based diagnostics at the time of survey, of which five used RDTs and three microscopy. Six health facilities had the first-line anti-malarial drug in stock at the time of survey.

A total of 20 of 114 (18%) health workers were interviewed. Twelve (60%) had clinical training background and eight non-clinical. The clinicians included two assistant medical officers (AMO) with advanced diploma in clinical medicine, nine clinical officers (CO) with diploma and one certified assistant clinical officer (ACO). The non-clinicians included three nurse midwives, three public health nurses, one nurse assistant and one medical attendant. Health workers with clinical background were responsible for 157/195 (81%) of the prescriptions. The main reasons given for involvement of non-clinicians in prescribing were shortage or absence of clinicians.

Eighteen (90%) of the health workers reported that they had been trained previously on RDT use. The training ranged from one to five days and included causes of fever, symptoms of malaria, performance and interpretation of RDT as well as management of patients with positive and negative RDT results.

A total of 195 patients, including 115 (59%) females and 80 males (41%), or their care takers/guardians in case of children, underwent exit interviews. Some 87 (45%) were children under five years of age. All patients presented with fever or history of fever. Other common symptoms included cough/chest symptoms, cold symptoms, headache and malaise (Table 1). Cold symptoms, diarrhoea and skin rash were more frequent in children under five years, while headache and malaise were more frequent in those above five years of age. Eighty patients had a diagnosis recorded in the CRF. The most commonly recorded diagnosis was malaria (54%), followed by respiratory tract infection (10%), skin/wound infections (8%) and genital/urinary tract infection (6%).

**Table 1:** Patients’ presenting symptoms (%)

**Variable**	**<5 years**	**>5 years**	**All patients**	**p-value**
Number of patients	87	108	195	
Fever	87 (100)	108 (100)	195 (100)	
Cough/chest symptoms	41 (47.1)	48 (44.4)	89 (45.6)	0.71
Flu-like symptoms	36 (41.1)	15 (13.9)	51 (26.2)	0.00
Headache	2 (2.3)	35 (32.4)	37 (19)	0.00
Generalized body malaise	1 (1.1)	20 (18.5)	21 (10.8)	0.00
Diarrhoea	15 (17.2)	7 (6.5)	22 (11.3)	0.02
Vomiting	13 (14.9)	8 (7.4)	21 (10.8)	0.09
Skin rash	13 (14.9)	6 (5.6)	19 (9.7)	0.03
Abdominal pain	4 (4.6)	13 (12.0)	17 (8.7)	0.07

### Malaria diagnosis and prescription practices

**Figure 1:**
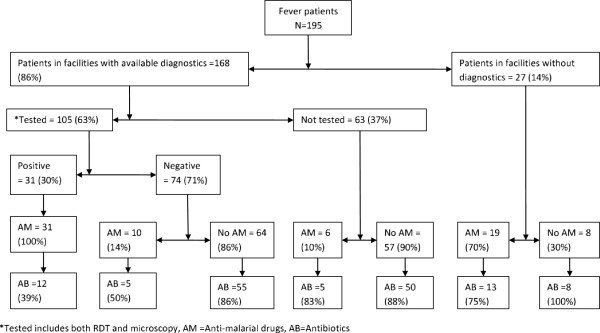
Testing rates and prescriptions of anti-malarial drugs and antibiotics.

One hundred and sixty-eight patients (86%) were seen in health facilities with available parasite-based diagnostics and 27 (14%) in facilities without testing opportunities (Figure 1). The overall parasite-based testing rate was 63% (105/168), i. e, 66% (53/80) in facilities equipped with RDT and 59% (52/88) in those with microscopy, and the overall malaria positivity rate was 30% (31/105). There were more positive cases in the RDT tested group than in the microscopy group (38 *vs* 21%, p = 0.05). Parasite-based testing was more common in children below five years of age than those above five years although positivity rates did not differ (Table 2).

**Table 2:** Parasite-based malaria testing and prescription rates (%) with anti-malarial drugs by age group

**Patient category**	**<5 years**	**>5 years**	**All patients**	**p-value**
Number of patients	87	108	195	
Patients tested for malaria	57 (65.5)	48 (44.4)	105 (53.8)	0.01
Patients with positive results	17 (29.8)	14 (29.2)	31 (29.5)	0.94
Patients with negative results	40 (70.2)	34 (70.8)	74 (70.5)	1.0
Malaria positive patients prescribed anti-malarial drugs	17 (100)	14 (100)	31 (100)	1.0
Malaria negative patients prescribed anti-malarial drugs	5 (12.5)	5 (14.7)	10 (13.5)	0.78
Non-tested patients	30 (34.5)	60 (55.6)	90 (46.2)	0.01
Non-tested patients prescribed anti-malarial drugs	6 (20.0)	19 (31.7)	25 (27.8)	0.24

Anti-malarial drugs were prescribed to all 31 (100%) patients with positive RDT/microscopy results and to 10/74 (14%) of negative patients (Figure 1). Among non-tested patients, anti-malarial drugs were given to 25/90 (28%), with a higher prescription rate in facilities without parasite-based diagnostics. Due to stock-outs of ACT in some of the facilities, 18% of patients treated with anti-malarial drugs were prescribed non-ACT (quinine or sulphadoxine-pyrimethamine). Among the 66 patients that received anti-malarial drugs, 35 (53%) were also prescribed antibiotics, whereas 113 (88%) of patients without an anti-malarial prescription were given antibiotics. Only 15 patients (8%) received prescriptions that did not include an anti-malarial drug or an antibiotic. There was no significant difference in the prescription of antibiotics between children under five years of age and older children and adults (80 *vs* 72%, p = 0.12). Prescription of antibiotics was higher among patients who tested negative for malaria than those with positive results (81 *vs* 39%, p < 0.01), and among those not tested compared to those tested for malaria (84 *vs* 69%, p = 0.01). Antipyretics were given to 159 (82%) patients either alone or in combination with anti-malarial drugs and/or antibiotics. Eighty-two patients (42%) were also prescribed other drugs such as anti-helminths, multivitamins, anti-fungals, iron supplements, etc.

Adherence to results of diagnostic tests was described as a situation where the patient was tested, had positive results and was treated with anti-malarial drugs; or the patient had negative results and was not treated with anti-malarial drugs, and overall adherence was 90.5%. In a multivariate analysis where several factors were included (health worker category, presence of vomiting, diarrhoea, headache, cough/chest pain and malaise), the model was able to account for 30% of adherence, and presence of vomiting and headache had significant contribution with an estimated odds ratio of 11.4 (95% Confidence interval (CI): 1.65-71.5) and 6.4 (95% CI: 1.1-35.8), respectively. The other factors were not statistically significant although removing them from the model lowered its contribution in adherence to 20%, suggesting that they are probably confounding factors.

A review of routine health facility records for the previous 12 months revealed that data were not compiled on a regular basis. In facilities with complete data, testing rates were high during the low transmission seasons (>80%) and varied considerably during high transmission seasons (15-100%). The proportion of over-prescription with anti-malarial drugs was higher than observed in the cross-sectional survey.

### Perceptions of health workers and patients about malaria diagnosis and treatment

Twenty health workers were interviewed, and according to them the main advantages of using RDTs were ease of use (14/20; 70%), taking a shorter time to produce results (11/20; 55%), and no need for electricity (6/20; 30%). Other advantages included helping to target treatment, confirm/rule out malaria, accurate/specific and patient satisfaction. The main disadvantage mentioned was false negative/inaccurate results (14/20; 70%) and others included lack of trust by patients, test remaining positive after treatment, tests not able to quantify parasites, negative results in patients with severe symptoms and invalid results (control band non-reactive).

Table 3 presents responses by the 18 health workers previously trained on RDT use to selected questions asked in the interview. The majority of health workers (13/18; 72%) had confidence in RDT results. Those who had confidence gave the following reasons: RDTs have been approved by relevant authorities, they are sensitive/specific, the results are consistent with symptoms, RDT results match transmission season and positive patients respond to anti-malarial drugs. Conversely, those who did not have confidence in RDTs mentioned false negative results, negative results in patients with symptoms, test remaining positive after treatment and good response to anti-malarial treatment in patients with negative results as their reasons. Seven (39%) health workers said patients do not trust RDT results and gave reasons, such as patients assuming that any fever is due to malaria (six), positive results reported at private health facilities (four) and suspecting they are tested for HIV (one).

**Table 3:** Health workers’ (HW) attitudes towards RDTs and prescriptions of anti-malarial drugs

**Questions addressed to health workers**	**Clinical**	**Non clinical**	**All HW**
	**N**	**N**	**N**
**Do you have confidence in RDT results?**			
Yes	8	5	13
No	3	2	5
**Do you sometimes prescribe anti-malarial drugs to patients with negative results?**			
Yes	7	5	12
No	4	2	6
**Do patients sometimes demand anti-malarial drugs when results are negative?**			
Yes	10	3	13
No	1	4	5
**Do patients trust RDT results?**			
Yes	7	4	11
No	4	3	7

When asked if they sometimes prescribe anti-malarial drugs to RDT negative patients, 12/17 (67%) of the health workers admitted to such practice. Eleven of these said that the rationale for prescribing anti-malarial drugs to RDT-negative patients was because the clinical symptoms were suggestive of malaria and one mentioned persistent symptoms in patients with negative results. Five health workers said patients sometimes demand anti-malarial drugs despite negative results, although only one admitted to prescribing due to patient demand.

Regarding knowledge about RDTs from the patient/caretaker perspective, 84 (43%) indicated that they had heard about RDTs, of whom 80 (95%) reported the health facility to be the main source of information. Overall 100% of patients with positive and 92% with negative RDT results said they were satisfied with the results. Trusting the health worker and the patient getting better after treatment were the main reasons for patient satisfaction, and hence trust of RDT results. Those not satisfied reasoned that they did not expect negative results and they were given positive results at a private dispensary.

Patients’ views of the advantages of using RDT included being fast, being able to confirm the diagnosis and producing reliable results. Other advantages were related to ease of use of the test, mainly the fact that there is no need for electricity or equipment, making it possible to use even in peripheral health facilities.

## Discussion

One year after introduction of RDTs as parasite-based malaria diagnostic tool in Kibaha District, Coast Region, Tanzania, this study found low testing rates, stock-outs of both RDTs and ACT, and non-adherence to negative test results.

Relatively, low testing rates were found in health facilities with parasite-based diagnostics, which was more pronounced in older patients than in children below five years of age. Stock-outs of RDTs together with staff shortage were reported as reasons for not using RDTs. In a study in Kenya, partial deployment of RDTs was said to lead to low testing rates of below 30%, although the rates did not exceed 54% even when testing facilities were available [14]. Another study reported under-use of testing facilities especially among older children and adults, which was unexpected as the previous recommendation was to treat all fevers in under-fives as malaria and test those above five years of age [18]. Lack of standard criteria for those who should be tested was also identified as responsible for the low testing rates and non-utilization of results [20]. New guidelines require testing of all patients suspected to have malaria, irrespective of age, after it was established that withholding anti-malarial drugs from parasite-negative children is safe [23-25].

Prescription of anti-malarial drugs for patients with negative parasite-based test results and those not tested is still practiced in areas where RDTs have been introduced. These two practices are contrary to the recommendations by WHO, which require universal testing of all patients suspected to have malaria and treatment with anti-malarial drugs be confined to parasitologically confirmed cases [5,7]. In other areas health workers have also continued prescribing anti-malarial drugs to RDT-negative patients [11,20,26,27]. However, the rate of over-prescription observed in this study is much lower than in earlier studies and corresponds better with rates observed in Tanzania [15,17]. Lower rates could be a reflection of experience and acceptance of RDTs, and better training and implementation of the RDT programme.

Stock-outs of ACT defined as running out of ACT for some periods before the next supply is received were also observed in some health facilities, which could be due to overuse of ACT in the treatment of patients with negative RDT results, or health system factors such as delays in procurement or underestimating requirements. This led to the prescription of non-ACT, contrary to national guidelines advocating the use of ACT as first-line anti-malarial drugs [28].

The majority of health workers had been trained on RDT use and in general health workers had good understanding of RDTs although a few mentioned that RDTs are sensitive and specific and improve rational use of drugs. Their non-adherence to test results could be due to prior training and experience, which leads to overemphasis on malaria, and limited capabilities to make alternative diagnosis. Similar findings have been reported in Tanzania and Cameroon [21,29] where justification given for treating patients with negative results are based on previous guidelines and experience with the use of anti-malarial drugs. In other studies, while health workers reported that RDT results were reliable, very few used them in case management, leading to overprescription among RDT-negative patients [12,20]. Previous malaria diagnosis and treatment guidelines stated that a negative test result does not rule out malaria, so clinical judgement should be used [28,30]. Proper training of health workers and equipping health facilities with the capacity to manage patients with both positive and negative RDT results will ensure that health workers have the capacity to identify and treat non-malarial causes of fever, an important factor in adherence to results [7].

Although a few health workers highlighted that sometimes patients with negative results demand anti-malarial prescriptions, the majority of patients trusted the results, mainly because they trust the health workers and get better after treatment, making it unlikely that they will demand anti-malarial drugs. A similar finding was reported in north-eastern Tanzania [22] where patients were not observed to demand anti-malarial prescription.

Having been given positive test results in private dispensaries was mentioned by both health workers and patients as a reason for lack of trust. This was observed in peri-urban areas where patients consult private clinics first, and go to government facilities for subsidized drugs when results are positive (personal observation). Informal discussions with health workers show that private health facilities give positive results to justify the money paid by patient for testing, to create an opportunity to sell anti-malarial drugs to the patient and, in the case of incompetent laboratory personnel, to avoid the danger of missing a true malaria infection. In the Cameroon study [29], health workers reported to give positive results to please the patient so that they feel they have not wasted their money on a test, as well as safeguard their reputation as a negative result would mean the health worker is incompetent.

It was also observed that prescription of antibiotics was higher among patients with negative results and those not tested. The rates observed in this study are higher than those reported in a previous study in the same area and in Zanzibar [15,31] but similar to those reported in another study [17]. Whether the antibiotic prescriptions were justified or not is outside the scope of this study. Still, there is a raising concern that the use of malaria diagnostics could lead to overprescription of antibiotics among patients with negative test results due to the lack of diagnostics and insufficient understanding of aetiologies of non-malarial fevers. Training of health workers in malaria diagnosis using RDTs should include training on identification of other causes of fever, including for example the provision and use of simple devices such as the respiratory rate timers for pneumonia used in integrated community case management (iCCM) in Uganda [32].

### Limitations

The sample size for the health workers’ interview was too small to allow further analysis of associations between variables such as education of health worker and prescription practice. The data collection method used, i. e, interviews with open-ended questions, did not offer an opportunity for cross-tabulating the responses provided. In-depth interviews could have provided a better assessment of perceptions and attitudes of both health workers and patients. The relatively good performance of health workers could partly be due to the Hawthorne effect. The availability of health facility records showing prescription practice over a longer period of time could allow validation of data obtained from exit interviews, when health workers knew that their prescription practices were being assessed. However, the value of such data was limited due to poor record keeping as seen in this study. Despite these limitations, the use of data from multiple sources including patients, health workers and health facility inventory allowed for a general assessment of prescription practices.

## Conclusion

Findings from this study show that overprescription with anti-malarial drugs is still practiced in an area of Tanzania where universal testing with RDTs have been introduced as official policy since treatment of RDT-negative patients and treatment based on clinical diagnosis without testing remains. The use of malaria diagnostics was also associated with higher prescription of antibiotics among patients with negative test results. Factors responsible for these practices include system factors such as non-availability of testing facilities, limited capacity to diagnose other causes of fever, staff shortage, and health workers perceptions about the importance of malaria and test results. In order to make RDTs have an impact in reducing unnecessary use of anti-malarial drugs and improve patient management, there is a need to address the identified factors by ensuring regular supply of testing facilities, retraining of health workers on RDTs and the importance of adhering to test results, training and support in the diagnosis and management of other causes of fever and close supervision.

## Competing interests

The authors declare that they have no competing interests.

## Authors’ contributions

MM conceived and designed the study, arranged and supervised the field work, analysed the data, and drafted the manuscript; BN, DK, ZP, AB and AM gave inputs to the study design; DK and SP helped with analysis and interpretation of data. All authors contributed in reviewing of the manuscript and approved the final version of the article.
